# Dopaminergic Modulation of Biological Motion Perception in patients with Parkinson’s disease

**DOI:** 10.1038/s41598-017-10463-2

**Published:** 2017-08-31

**Authors:** Tingting Liu, Panpan Hu, Ruihua Cao, Xing Ye, Yanghua Tian, Xianwen Chen, Kai Wang

**Affiliations:** 1 Department of Neurology, the First Affiliated Hospital of Anhui Medical University, Hefei, Anhui Province China; 2 Anhui Province Key Laboratory of Cognition and Neuropsychiatric Disorders, Hefei, Anhui Province China; 3 Collaborative Innovation Center for Neuropsychiatric Disorders and Mental Health, Hefei, Anhui Province China; 4 Department of Geriatric Medicine, Anhui Provincial Hospital, Hefei, Anhui Province China; 5 Department of Neurology, the Affiliated Drum Tower Hospital of Nanjing Medical University, Nanjing, Jiangsu Province China; 6 Department of Medical Psychology, Anhui Medical University, Hefei, Anhui Province China

## Abstract

Parkinson’s disease (PD) is a progressive neurodegenerative disorder pathologically characterized by a selective loss of dopaminergic neurons in the substantia nigra. In previous studies, greater attention was paid to impairments in motor disturbances in contrast to impairments of cognitive function in PD that was often ignored. In present study, a duration discrimination paradigm was used to assess global and local biological motion (BM) perception in healthy controls(HCs) and PD patients with and without dopamine substitution treatment (DST). Biological motion sequences and inanimate motion sequences (inverted BM sequences) were sequentially presented on a screen. Observers were required to verbally make a 2-alternative forced-choice to indicate whether the first or second interval appeared longer. The stimuli involved global and local BM sequences. Statistical analyses were conducted on points of subjective equality (PSE). We found significant differences between untreated PD patients and HCs as well as differences between global and local BM conditions. PD patients have a deficit in both global and local BM perception. Nevertheless, these two BM conditions can be improved under DST. Our data indicates that BM perception may be damaged in PD patients and dopaminergic medication is conducive to maintain the BM perception in PD patients.

## Introduction

Biological motion (BM) perception is a multi-level phenomenon that requires bottom-up integration of signals from basic visual motion perception along with top-down social cognition^1^. It consists of both global and local processes. While the global process is informed by the display’s spatiotemporal organization^2^, the local process relies predominantly on the motion signals of the individual dots^3, 4^. Studies of the visual perception of BM generally use point-light stimuli that are created by attaching point-lights to a person’s body and head and then recording the person’s movements so that only the point-lights are visible^5^. These stimuli are easily manipulated and typical observers’ can readily gather considerable social information including the actor’s identity^6, 7^, gender^8^, actions^9^, emotion^10, 11^, and intentions^12^. However, when the point-light displays are inverted, the perception of BM is strongly impaired. This phenomenon is called the “inversion effect”^13–15^and it is caused by an impairment in the spatial configuration of BM sequences. In addition, there is a second inversion effect that relies on local motion^16^.

Recently, Jiang and colleagues showed that BM signals could prolong their perceived temporal duration, independent of global configuration and without the observer’s subjective awareness of their biological nature^17^. They adopted a duration discrimination paradigm and found that an upright BM sequence was perceived for a significantly longer period of time compared to the inverted, inanimate sequence of the same physical duration. This temporal dilation could be extended to spatially scrambled biological sequences that only involve information from local biological motion. However, this effect completely disappears when critical BM characteristics are removed. This additional control experimental sequence might suggest that the differences in temporal dilation found between groups were specific to BM signals. Studies have shown that observers with lesions in the human premotor or motor system are impaired in their ability to perceive human movements^18, 19^. PD patients are typically impaired in movement execution. The dopaminergic cells in the basal ganglia system are also heavily involved in motor programming processes, such as the preparation, initiation, and execution of movement and also for maintaining a readiness for action^20^. Thus, PD provide an ideal model to explore the BM perception.

Parkinson’s disease (PD) is a neurodegenerative disease characterized by tremor, rigidity, bradykinesia, and postural abnormalities and has led to a significant loss of mobility in daily life. The frequency of the disorder is about 1.3 cases per 100,000 people younger than 45 years of age, 3,100 per 100,000 in those aged 75–85 years, and 4,300 per 100,000 in those older than 85 years^21^. It is well accepted that PD is caused by the depletion of dopamine (DA)-producing neurons in the substantia nigra pars compacta^22^. Besides motor impairment, dopamine depletion can also result in cognitive impairment and social behavior disorder, even in early-stage disease without dementia^23, 24^. Accumulating evidence suggests that individuals with PD eventually develop impairments, not only in memory and visuospatial function, but also in attention and executive function^25–27^. Moreover, PD patients have been found to show deficits in visual scanning abilities, “theory of mind” (TOM)^28^ and facial emotion recognition^29^. It is generally argued that disorders of facial emotion recognition in PD result from a loss of dopaminergic neurons leading to dysfunctional frontosubcortical systems^30–32^.

Cao and colleagues found that temporal dilation effect was significantly reduced for PD patients in both intact and scrambled BM conditions^33^. Besides, a new research suggested that PD patients have deficit in perceiving biological motion, which is independent of gait dysfunction and low-level vision changes^34^. Thus, we think that there is a BM perception deficit in PD. But, whether the dopaminergic transmitter system are also involved in the BM perception is remain unknown. In the current study, we have used the duration discrimination paradigm to assess the effects of animate motion signals on time perception. Time perception was used as an implicit measure of the dynamic properties of apparent human movement. We applied two duration discrimination tasks to compare temporal expansion effect of BM signals between healthy controls and PD patients (with and without dopamine substitution treatment (DST)). In two tasks, intact and scrambled point-light walkers, with their inverted stimuli as opposite inanimate motions, were presented sequentially. The intact BM stimulus contains local motion signals and global form signals, while the scrambled stimulus contains only local motion signals. Here, we investigated the potential role of dopamine neurotransmitter system in BM perception in PD patients and HCs.

## Results

### Demographic Data, PD-Related clinical characteristics, and Neuropsychological Findings

There were 25 PD patients (16 male and 9 female) and 25 healthy controls (19 male and 6 female) participating in the study in total. As normally distributed data, age (t (48) = 0.55, *P* > 0.05, by independent samples *T*-tests) and educational (t (48) = 0.40, *P* > 0.05, by independent samples *T*-tests) levels were not significantly different between the two groups. As a non-normally distributed data, gender was not significantly different between the two groups too (Z = 0.92, *P* > 0.05, by Mann-Whitney U tests) (see Table 1).

**Table 1:** Demographic Data, Clinical characteristics and Neuropsychological Findings of PD and HCs (Mean ± Standard Deviation).

	PD	HCs
Number	25	25
Age (years)	61.96 ± 7.92	60.60 ± 9.49
Gender (M/F)	16/9	19/6
Education Background (year)	7.76 ± 4.24	7.32 ± 3.60
Disease duration (years)	3.70 ± 1.93	—
onset side (left/right)	14/11	—
Hoehn and Yahr stage		
Stage 1.5	8	—
Stage 2.0	10	—
Stage 2.5	4	—
Stage 3.0	3	—
MMSE score (out of 30)	27.92 ± 1.50	27.56 ± 1.73
VFT^*^	11.56 ± 1.58	13.12 ± 2.19
DS(f)^*^ (out of 8)	5.56 ± 0.71	6.12 ± 0.93
DS(b)^*^ (out of 7)	3.52 ± 0.65	4.20 ± 0.87

PD = Parkinson’s Disease; HCs = Health controls; DS (b) = Digital Span (backward); VFT = verbal fluency task; DS (f) = Digital Span (forward); MMSE = mini-mental state examination. *Indicates a significant effect of group (p < 0.05).

As normally distributed data, the MMSE scores did not vary significantly between the two groups (t (48) = 0.79, *P* > 0.05, by independent samples *T*-tests), while VFT (t (48) = 2.89, *P* < 0.05, by independent samples *T*-tests) scores were significantly different between the two groups. As non-normally distributed data, the DS (DS [f] (Z = 2.38, *P* < 0.05, by Mann-Whitney U tests) and DS [b](Z = 3.05, *P* < 0.05, by Mann-Whitney U tests)) scores were significantly different between the two groups (see Table 1).

Disease severity was assessed using the Hoehn–Yahr Scale^35^, a commonly used system for describing how the symptoms of PD progress and the relative level of disability, ranging from stages, 1–5.

A paired-sample *T*-test revealed that the UPDRS III scores were significantly different between the PD patients with (22.76 ± 7.65) and without (29.48 ± 8.24) DST (t (24) = 2.99, *P* < 0.05).

### Duration discrimination task findings between the PD and control groups

**Figure 1:**
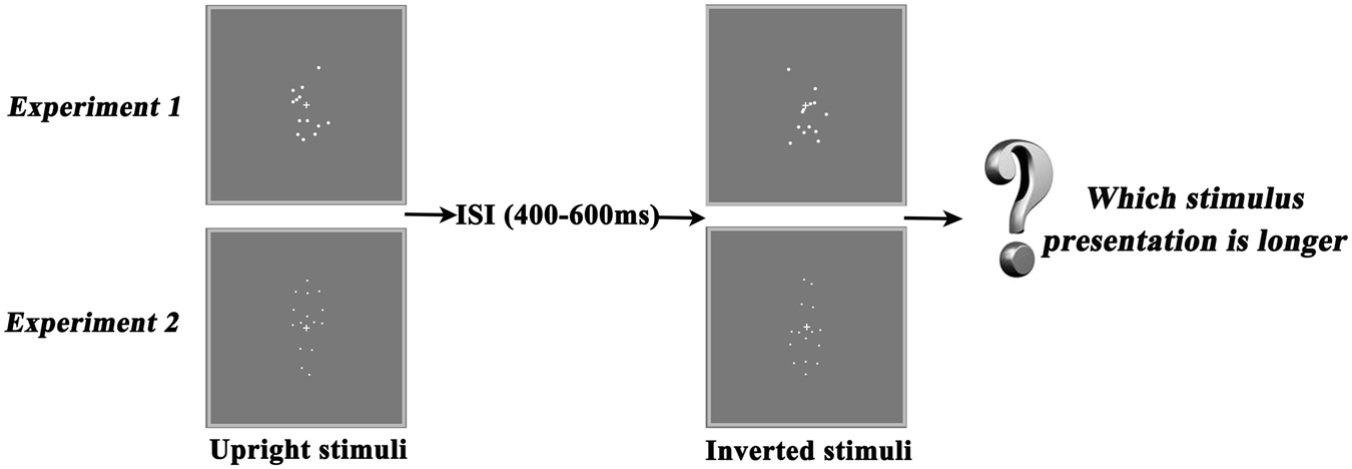
Scrambled point-light walkers, and intact point-light walkers were used in the present study, including upright and inverted stimuli.

The PSEs and DLs for PD patients (with and without DST) and HCs in the intact and scrambled BM conditions are shown in Table 2 (they are all normally distributed data). Figure 1 represents the task used in the present study. The results of PD patients and HCs from this duration discrimination task were fitted with a Boltzmann sigmoid function (Equation 1).

**Table 2:** Point of subjective equality (PSE) and difference limen (DL) for Parkinson’s disease(with and without DST) (PD; n = 25) and HCs (n = 25) across intact and scrambled BM conditions (Mean ± Standard Deviation).

Condition	Group	PSE	DL
Intact BM	PD without DST	−0.31 ± 97.60^*^	542.73 ± 202.32
	PD with DST	−99.97 ± 81.46	532.26 ± 205.74
	HCs	−88.92 ± 78.09	630.16 ± 247.80
Scrambled BM	PD without DST	−14.18 ± 51.63^*^	681.17 ± 319.02
	PD with DST	−54.72 ± 78.40	664.06 ± 275.17
	HCs	−59.53 ± 56.28	672.69 ± 318.46

*In comparison with PD without DST and HCs: P < 0.05.

**Figure 2:**
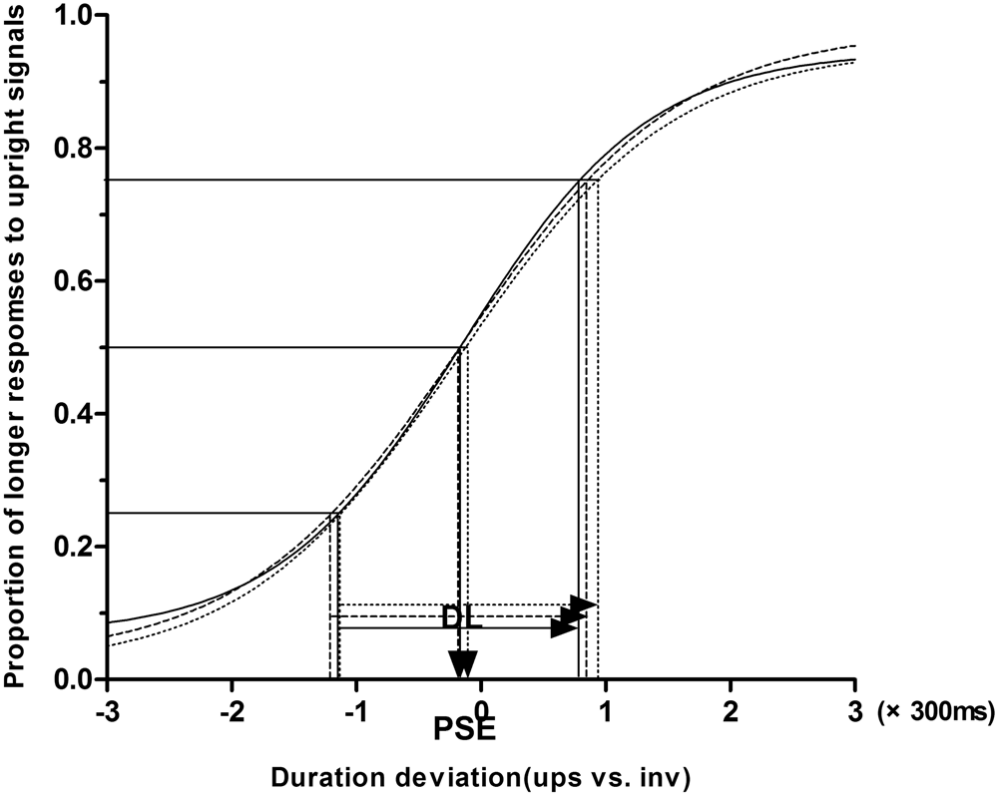
Psychometric function for observers in scrambled BM condition with a standard duration of 1000 ms. The vertical solid-line arrow and long dash-line arrow show a significant negative PSE in HCs and PD patients with regular DST. However,the vertical short dash-line arrow shows there was no significant negative PSE in PD patients without DST. The horizontal arrows indicate the DL.

**Figure 3:**
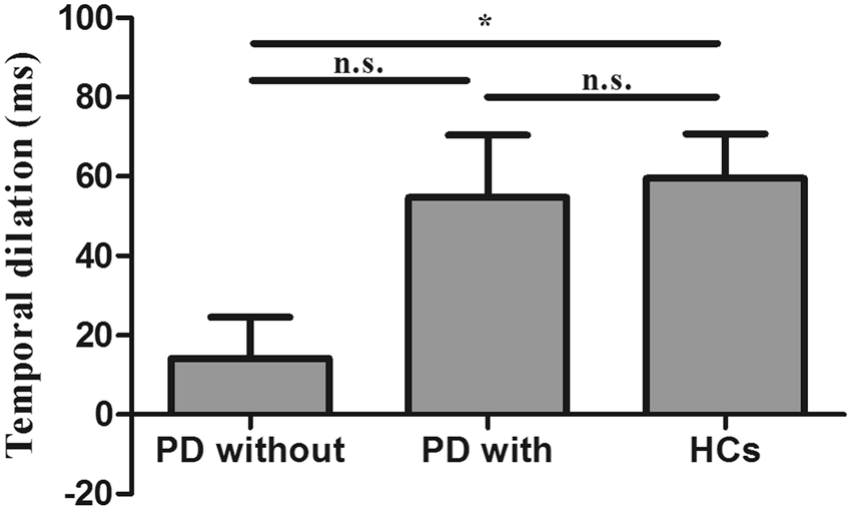
Duration discrimination results from experiment 1. The temporal dilation effect (i.e., minus PSE) of the upright biological motion stimuli was significantly larger than that of the inverted biological motion stimuli. The PSE of the scrambled biological motion stimuli in PD without DST and HCs were significantly different, but the PSE in PD with and without DST were not significantly different (*P < 0.05; n.s., not significant). Error bars show standard errors.

In experiment 1 for the scrambled biological motion condition, both the PD patients and HCs reported that they were unaware of a human figure (upright or inverted) when observing the stimuli. A one-sample *t*-test revealed a significant negative PSE in HCs (t (24) = −5.29, *P* < 0.001), indicating that the temporal dilation effect of BM stimuli can be induced, on its own accord by the local motion signals. That is to say, the temporal dilation effect was caused by the BM signals rather than the familiarity of the upright global figures. For PD patients without DST, there was no significant negative PSE (t (24) = −1.37, *P* = 0.182), suggesting that temporal dilation effect of scrambled BM stimuli was impaired in PD patients. However, we were surprised to find that there was a significant negative PSE in PD patients with DST. One-way ANOVA revealed significant differences between PD patients without DST, PD patients with DST and HCs (F (2, 72) = 3.89, *P* < 0.05). There were also significant differences between PD patients without DST and HCs (*P* < 0.05), and between PD patients with and without DST (*P* < 0.05). There was no significant difference between PD patients with DST and HCs (*P* = 0.79). After Bonferroni correction, there was significant differences between PD without DST and HCs (*P* < 0.05), but no significant difference between PD with and without DST (*P* = 0.08). Moreover, the observers’ temporal discrimination sensitivities (difference limen) were not significantly different between the three groups (F (2, 72) = 0.02, *P* > 0.05, by One-way ANOVA). The fitted summary of psychometric functions are shown in Fig. 2. The temporal dilation effect are shown in Fig. 3.

**Figure 4:**
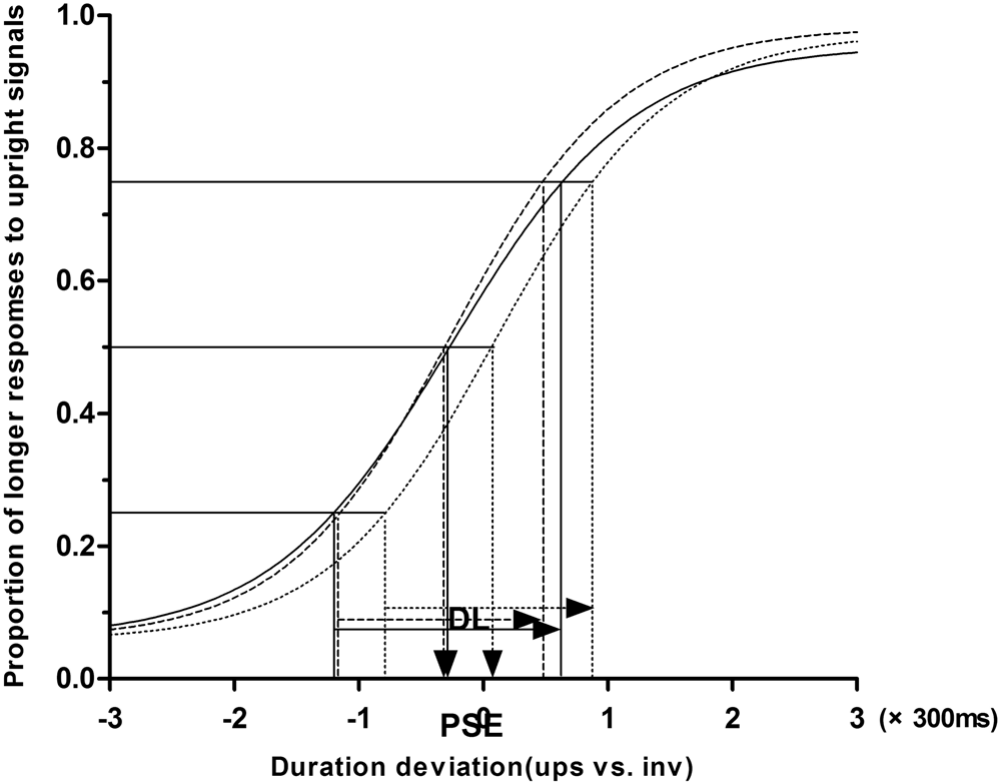
Psychometric function for observers in intact BM condition with a standard duration of 1000 ms. The vertical solid-line arrow and long dash-line arrow show a significant negative PSE in HCs and PD patients with regular DST. However,the vertical short dash-line arrow shows there was a positive PSE in PD patients without DST. The horizontal arrows indicate the DL.

**Figure 5:**
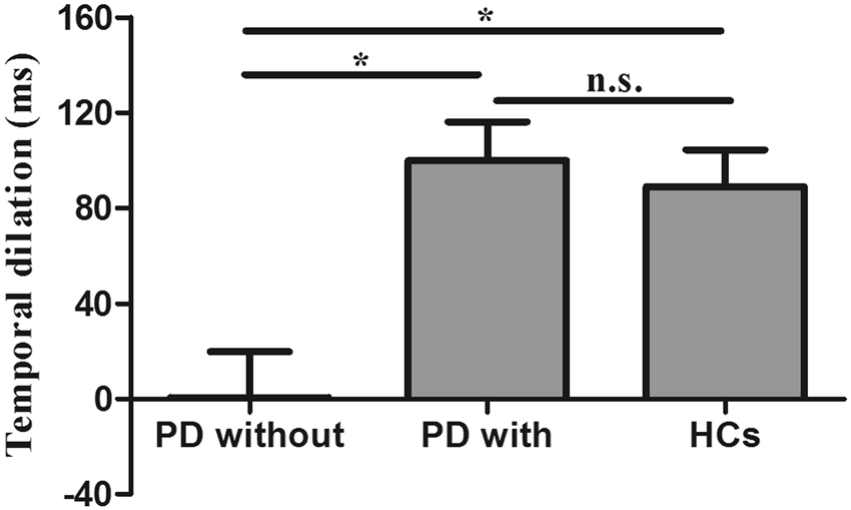
Duration discrimination results from experiment 2. The temporal dilation effect (i.e., minus PSE) of the upright biological motion stimuli was significantly larger than that of the inverted biological motion stimuli. The PSE of the intact biological motion stimuli in PD without DST and HCs were significantly different, the PSE in PD with and without DST were also significantly different (*P < 0.05; n.s., not significant). Error bars show standard errors.

In experiment 2 for the intact biological motion condition, PD patients and HCs reported that they could recognize both the inverted and upright walking human figures when observing the stimuli. A one-sample *t*-test revealed a significant negative PSE in HCs (t (24) = −5.69, *P* < 0.001), suggesting the presence of a temporal dilation effect for the upright intact BM stimuli compared to the inverted figure of identical temporal duration. For PD patients without DST, there was no significant negative PSE (t (24) = −0.02, *P* = 0.988), suggesting that temporal dilation effect of intact BM stimuli was impaired in these patients. In contrast, we found that there was a significant negative PSE in PD patients with DST. One-way ANOVA revealed significant differences between PD patients with and without DST and HCs (F (2, 72) = 10.06, *P* < 0.001). There were also significant differences between PD patients without DST and HCs (*P* < 0.01) and between PD patients with and without DST (*P* < 0.001). There was no significant difference between PD patients with DST and HCs (*P* = 0.65). After Bonferroni correction, there were significant differences between PD without DST and HCs (*P* < 0.01) and between PD with and without DST (*P* < 0.001) too. Moreover, the observers’ temporal discrimination sensitivities (difference limen) were not significantly different between the three groups (F (2, 72) = 1.5, *P* > 0.05, by One-way ANOVA). The fitted summary of psychometric functions are shown in Fig. 4. The temporal dilation effect are shown in Fig. 5.

### Correlation analysis

To determine whether the PSEs of the PD patients both on and off DST were related to any psychosocial factors, we used Pearson correlation analyses and found that PSEs were not related to age, disease duration, Hoehn and Yahr stage, or MMSE, VFT, DS (DS (f) and DS (b) (all *P* > 0.05, see Table 3).

**Table 3:** The correlation between PD patients (both with and without DST) with various factors.

Correlation		Age		Disease duration		H-Y stage		MMSE		VFT		DS(f)		DS(b)	
		r	*P*	r	*P*	r	*P*	r	*P*	r	*P*	r	*P*	r	*P*
PSE (Intact BM)	on	−0.01	0.98	−0.24	0.25	0.03	0.90	0.13	0.54	−0.01	0.97	0.19	0.36	0.09	0.66
	off	0.07	0.75	−0.05	0.80	−0.06	0.77	0.39	0.06	−0.07	0.73	0.22	0.30	0.05	0.83
PSE (Scrambled BM)	on	0.24	0.26	0.24	0.24	0.24	0.26	0.27	0.20	−0.06	0.78	0.14	0.50	0.05	0.82
	off	0.28	0.17	0.32	0.12	0.33	0.11	0.19	0.37	−0.28	0.17	−0.17	0.41	−0.13	0.55

“on” stands for with DST; “off” stands for without DST.

## Discussion

The results of this study showed that there is a negative PSE for both the global BM signals and local ones in PD patients with regular DST and healthy controls. However, the PSE of PD patients without DST (after overnight withdrawal of dopamine substitution treatment) for both global and local biological motion signals were either positive or close to a positive. Our results showed that a negative PSE indicated temporal dilation in healthy controls and PD patients with DST, an effect confirmed by Wang and Jiang^17^. Specifically, the BM signals implicated in the stimulus prolonged their perceived temporal duration.

Although the PSE of PD patients with DST for local BM signals have no statistic difference with PD patients without DST. There is a relative negative PSE of PD patients with DST for local BM signals. Thus, we inferred from these observations that PD patients showed deficits in both global and local BM perception, whereas these BM perception deficits can be restored by dopamine administration. Our results agrees with a previous study that demonstrated impairments in perception natural movements relative to unnatural movements in PD. Their perception of unnatural movements is not different from healthy controls^36^. We suspected that the observed effect from our study was probably specific to BM signals.

The definition of the BM perception is an observer recognizing a biological entity executing a decipherable activity^37^. There is a perceptual advantage of perceiving human biological motion compared to non-biological motion^38^, an increased BOLD response to an upright as compared with an inverted walker was also reported over the frontal cortices^39^, which lends strong support to the study of Wang and Jiang^17^. That is, relative to the inverted walker, the upright walker which carried BM information generated increased response over frontal cortices. A lot of studies have found that the posterior superior temporal sulcus (pSTS) plays a key role in the process of BM, other brain areas involved include premotor, inferior frontal region and so on^40, 41^. Among these, the mirror neuron system is one of the hotspots of research in the neuromechanism of BM perception. Mirror neuron system was first identified in area F5 of the monkey premotor cortex and it was shown to be activated by both executing and observing a particular action^42–44^. Studies depending on the electrophysiological methods (e.g. EEG) and brain imaging (fMRI, PET) support the existence of a mirror neuron network in the human brain^45^. A set of brain areas involving the inferior and superior parietal lobules, the anterior intraparietal sulcus, and the inferior frontal gyrus (IFG) has been dubbed the “mirror system” in humans^46^. Recently, local field potential (LFP) indicated that the basal ganglia was also involved in the activity of mirror neuron system^47, 48^. Given that point-lights BM sequence activated human mirror neuron system^46^, we suggest that basal ganglia maybe related to the BM perception.

The progressive degradation of the dopaminergic cells in the basal ganglia system is known to result in the occurrence of Idiopathic PD. Patients with PD show impairments in performing various cognitive tasks that primarily rely on the frontal lobes^49^. And dopamine depletion is explicitly linked to the dysfunction of prefrontal cognitive areas through three different frontostriatal circuits: the dorsolateral, orbital, and the anterior cingulate circuits^50^. Studies have shown that the basal ganglia system plays an active role in the motor planning processes that includes maintaining a readiness for action, preparing, initiating, and executing a particular action^20^. By administering the basal ganglia with dopamine, the information that used to perform a decisive action (such as reaching for a static object) has been successfully passed between cortical and subcortical structures^51^. Thus, we conclude that the frontal cortices, basal ganglia and dopamine were essential to the motor programming processes.

The main finding of our study is that there is a temporal expansion effect for BM signals in healthy controls and PD patients with regular DST. In addition, when PD patients went through an overnight withdrawal of dopamine substitution treatment (without DST), the temporal expansion effect were impaired. From these observations, we suggest that both BM perception and PD were highly related to the basal ganglia and frontal cortices. Our conclusions are consistent with the observed results.

In the present study, we applied the duration discrimination paradigm to test the effects of BM signals on time perception. Time perception was used as an implicit method to evaluate the dynamic properties of distinct human movement. Whether or not time perception was impaired in PD patients was remain controversial. While some studies claim that it was impaired^52, 53^, others don’t^54, 55^. Previous findings have suggested that the sub-second intervals are probably processed by a motor circuitry consisting of the primary sensorimotor cortex and cerebellum, the supra-second intervals more likely activate DLPFC and parietal cortices which are associated with cognitive functions^56^. Our experiment only involved sub-second time processing. From the above, we thought that PD patients may be not impaired in cognitive sub-second time processing. The duration discrimination test, used in the present study is easy to understood and there is no need for attention processes. Besides, we found that the observers’ temporal discrimination sensitivities (difference limen, DL) were not significantly different between PD patients (with and without DST) and HCs, which may support the speculation that deficits in time perception per se, were minimal in our experiments. The absence of a non-BM stimuli and a truly time perception paradigm are main limitation of our study.

To summarize, we conclude that PD patients have a deficit in BM perception (both global and local ones) and this deficit can be restored by administering regular dopamine substitution. These results provide evidence that basal ganglia and frontal cortices are all linked to BM perception and dopamine plays an important role in the processes of BM perception. Future studies should utilize neuroimaging techniques to further confirm the role of dopamine in the BM perception.

## Methods

### subjects

Twenty-five early to moderately affected patients with PD (16 males, 9 females; all right-handed) and twenty-five HCs (19 males, 6 females; all right-handed) participated in the study (see Table 1). PD patients were recruited from outpatients who were diagnosed and regularly treated at the First Hospital of Anhui Medical University, Anhui Province, China. All participants fulfilled Parkinson’s Disease Society Brain Bank clinical criteria for definite PD^57^. Disease severity was graded according to the motor score on Section III of the Unified Parkinson’s Disease Rating Scale (UPDRS III)^58^ and the Hoehn and Yahr rating scale^35^.Exclusion criteria for PD patients included: (1) patients who had a history of neurological or psychiatric illnesses other than PD, such as depression, cerebral infarction or migraine; (2) dementia based on clinical examination or a Mini Mental State Examination (MMSE) score ≤ 24^59^; (3) use of active central nervous system therapies other than levodopa, amantadine and dopamine agonists, alcohol or other substance abuse or dependence and, (4) deficits in vision and hearing. Exclusion criteria for the HCs included: (1) the presence of psychiatric or neurological illness, such as depression, cerebral infarction or migraine; (2) dementia on the basis of clinical examination or a MMSE score ≤ 24; (3) the use of medication with central nervous system effects. The study was approved by the Ethics Committees of Anhui Medical University and executed in agreement with the Declaration of Helsinki. All subjects provided written informed consent.

### Neuropsychological Assessment

The following neuropsychological tests were administered to all participants and compared between the PD and the HCs group: (1) the MMSE score measures global cognitive functions; (2) the Hamilton Depression Scale measures the presence of depressive states; (3) verbal fluency (number of words per minute) measured frontal functions; (4) the Digit Span test estimated short-term memory and executive functions including forward and backward spans.

### Experimental stimuli generation

Stimuli were generated and displayed using MATLAB (Mathworks) and the Psychophysics Toolbox extension^60^. The intact point-light BM videos were adopted from Vanrie and Verfaillie^61^. These videos were generated by videotaping the walking gait of an actor and then, encoding the joint positions in the digitized videos. In the scrambled BM sequences, where the local motion cues were preserved, the starting position of each point was randomly displaced from its vertical position about a central axis within a similarly sized region as the intact BM sequences. Only the global configuration information was entirely disrupted, so that the familiar limb sequences were harder to identify. Inverted BM counterparts (intact and scrambled) were derived by vertically mirror-flipping all the motion sequences.

### Experimental Procedure

Stimuli were white on a gray background and observers viewed them from approximately 80 cm away. In each trial, two stimuli (e.g., an upright and an inverted intact BM sequence) were sequentially presented in the center of the screen, such that the dots subtended approximately 4.0° × 6.8° in visual angle. One of the stimuli (upright or inverted figure) was randomly selected to be presented for 1000 ms; the other was displayed for 100 ms, 400 ms, 700 ms, 1000 ms, 1300 ms, 1600 ms, or 1900 ms, resulting in a total of 7 test conditions. Therefore, the difference between the presentation duration of the two stimuli (upright vs. inverted) was −900 ms, −600 ms, −300 ms, 0 ms, 300 ms, 600 ms, or 900 ms. Between the two stimuli displays, a blank interval with a randomized duration of 400–600 ms was inserted to avoid a potential interference effect. Furthermore, the presentation order of the two stimuli and the initial frame of the point-light display for each test stimulus was randomized across trials. Observers were required to verbally make a 2-alternative forced-choice to judge as accurately as possible which interval (first or the second) was longer, regardless of the type of stimulus shown. Participants were explicitly told not to count aloud or sub-vocally and that neither the stimulus order nor its content were predictive of the stimulus presentation duration. The next trial started only after observers made their choice for the previous one.

Experiment 1 adopted a scrambled BM sequence (upright and inverted) and experiment 2, an intact one (upright and inverted). In order to keep every observer naive as to the nature of the scrambled sequences, all the observers were assigned experiment 1 before initiating experiment 2, since the intact BM sequence might introduce the concept of a human figure and have an impact on the scrambled BM sequence. After completion of both experiments, each subject was asked what he or she could recognize from the sequences. Each experiment consisted of 70 trials, with 10 trials for each test condition so that each observer performed a total of 140 trials. A rest period was included, after every 20–30 trials.

PD patients performed the experiments in the presence of regular dopamine substitution treatment (DST) and in the absence of DST (after overnight withdrawal of DST). Patients were also clinically examined by a motor disorder specialist, according to UPDRS III. Initially, 12 of the PD patients were tested without DST subsequent to an examination with DST the following month. The experimental protocol was switched around for the rest of the 13 patients; DST first and then without DST the following month (see Table 4)^62^. Their regular DST was resumed after completion of the experiments.

**Table 4:** PD patients with and without DST in details.

Regular DST					Time since last medicine intake (h)
Levodopa + Benserazide (mg/d)	Other anti-parkinsonian medicine			L-dopa Equivalent Dose^62^ (mg/d)	
	DA agonist (mg/d)	MAOB inhibitors (mg/d)	Amantadine (mg/d)		
250	—	10 (selegiline)	300	600	16
500	50 (piribedil)	—	—	450	16
375	100(piribedil)	—	—	400	17
—	—	—	300	300	20
500	100(piribedil)	—	—	500	18
125	—	—	300	400	18
187.5	—	—	—	150	17
250	—	—	300	500	15
187.5	—	—	200	350	15
187.5	150(piribedil)	—	—	300	15
375	—	—	300	600	18
375	—	20 (selegiline)	200	700	15
250	—	—	—	200	16
625	—	—	300	800	15
375	—	—	200	500	18
250	—	—	300	500	15
500	100(piribedil)	—	300	800	15
250	50(piribedil)	—	—	250	17
125	—	—	200	300	18
187.5	—	—	—	150	16
500	—	—	300	700	18
187.5	—	—	200	350	24
500	0.75(pramipexole)	—	300	775	16
375	—	—	—	300	20
187.5	—	—	200	350	18

### Analysis

The results of each individual observer from this 2-alternative forced-choice task were fitted with a Boltzmann sigmoid function (Equation 1), in which the X-axis showed the difference between the resentation durations of the 2 stimuli (upright vs. inverted), ranging from −900 ms to + 900 ms and the Y-axis showed the proportion of the “long” responses to the upright stimuli. The statistical analyses were conducted for the point of subjective equality (PSE) and difference limen (DL). PSE refers to the point at which the observers’ perceived the 2 stimuli equal in terms of the presentation duration and it was estimated by the midpoint of the Boltzmann function:

1 $${\rm{f}}({\rm{x}})=1/{(1+\exp [({\rm{x}}-{\rm{x}}0)/{\rm{\omega }}])}^{5}$$

A negative PSE meant that when the observers’ perceived the 2 stimuli at the same duration time, the upright stimulus was presented for less time than the inverted counterpart in physical duration (i.e., temporal expansion), whereas a positive PSE indicated the reverse (i.e., temporal compression). DL was calculated by estimating the interquartile range of the fitted function, and it was used to measure the temporal discrimination sensitivity^63^ (In the Boltzmann function f(x) = 1/(1 + exp[(x  − x0)/ω]), x0 stands for PSE, w stands for DL). Figures 2, 4 depicts this function.

Statistical analyses were carried out with the SPSS software version 17.0 for Windows. The Kolmogorov-Smirnov test was used to determine the normality of the data. For normally distributed data, parametric tests were used (*t*-test for independent samples, analysis of variance with repeated measures: ANOVA). For non-normally distributed data, nonparametric tests (Mann-Whitney U tests) were used. To examine potential relations between variables in the PD group, we calculated Pearson correlations. The level of significance for all statistical tests was set at P = 0.05.
